# Neurodevelopmental Disorders Caused by Defective Chromatin Remodeling: Phenotypic Complexity Is Highlighted by a Review of ATRX Function

**DOI:** 10.3389/fgene.2020.00885

**Published:** 2020-08-11

**Authors:** Sara Timpano, David J. Picketts

**Affiliations:** ^1^Regenerative Medicine Program, Ottawa Hospital Research Institute, Ottawa, ON, Canada; ^2^Department of Biochemistry, Microbiology, and Immunology, University of Ottawa, Ottawa, ON, Canada; ^3^Department of Cellular and Molecular Medicine, University of Ottawa, Ottawa, ON, Canada; ^4^Department of Medicine, University of Ottawa, Ottawa, ON, Canada; ^5^University of Ottawa Brain and Mind Research Institute, Ottawa, ON, Canada

**Keywords:** intellectual disability, neurodevelopmental disorder, *ATRX*, ATR-X syndrome, chromatin remodeling, SWI/SNF

## Abstract

The ability to determine the genetic etiology of intellectual disability (ID) and neurodevelopmental disorders (NDD) has improved immensely over the last decade. One prevailing metric from these studies is the large percentage of genes encoding epigenetic regulators, including many members of the ATP-dependent chromatin remodeling enzyme family. Chromatin remodeling proteins can be subdivided into five classes that include SWI/SNF, ISWI, CHD, INO80, and ATRX. These proteins utilize the energy from ATP hydrolysis to alter nucleosome positioning and are implicated in many cellular processes. As such, defining their precise roles and contributions to brain development and disease pathogenesis has proven to be complex. In this review, we illustrate that complexity by reviewing the roles of ATRX on genome stability, replication, and transcriptional regulation and how these mechanisms provide key insight into the phenotype of ATR-X patients.

## Introduction

Neurodevelopmental disorders (NDD) are highly complex and heterogeneous conditions that have a global prevalence of approximately 2–3% of the population. Despite being aware of these conditions for over a century, it is only within the last decade that the development of exome and whole genome sequencing has dramatically enhanced the discovery of the underlying causes of these disorders. Indeed, the SysID database^1^ list 1,334 genes (updated March 26, 2020) that contribute to intellectual disability (ID) (Kochinke et al., 2016), while approximately 100 genes are associated with autism spectrum disorder (ASD) (Satterstrom et al., 2020). Interestingly, a substantial proportion of NDD causing genes are involved in chromatin and/or transcriptional regulation including the broad family of ATP-dependent chromatin remodelers.

**FIGURE 1:**
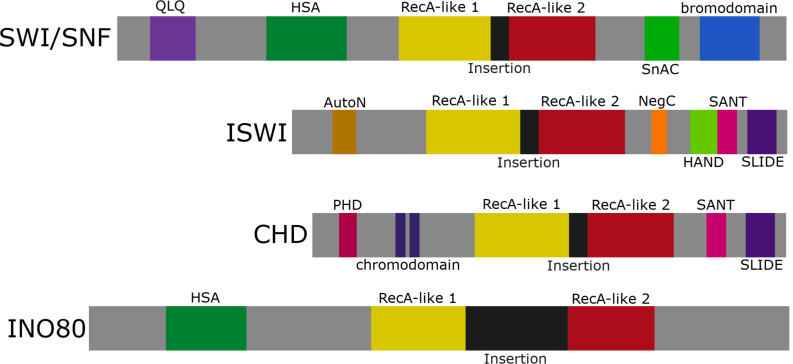
The ATP-dependent chromatin remodeling family. Representation of the four chromatin remodeling groups: SWI/SNF, ISWI, CHD, and INO80. Each group contains an ATPase domain subdivided into RecA-like lobes 1 and 2 separated by a variable linker region (labeled insertion). SWI/SNF and INO80 share an HSA domain, while ISWI and CHD share a SANT and SLIDE domain.

Chromatin remodelers utilize energy from ATP hydrolysis to alter nucleosome spacing/density or to facilitate histone variant exchange (Bowman and Poirier, 2015). There are four main families of ATP-dependent chromatin remodelers characterized by their conserved ATPase domain of the helicase II superfamily (Figure 1). These families are divided into the (1) SWI/SNF group, large complexes made up of ∼15 subunits, (2) ISWI group, heterodimers and four subunit complexes, (3) CHD group, complexes that incorporate up to ∼10 subunits, and (4) INO80 group, ∼15 subunit complexes. In addition, the focus of this review is ATRX which represents one of several orphan families that have been less studied mechanistically. In addition to the ATPase domain that is subdivided into two RecA-like lobes, these chromatin remodeling enzymes are characterized by additional motifs that facilitate protein–protein interactions (e.g., HSA and QLQ domains), DNA interactions (e.g., HAND and SLIDE domains), and chromatin interactions (e.g., SANT, chromodomain, and bromodomain) (Figure 1). The SWI/SNF and INO80 family primarily promote transcription and DNA repair by sliding/ejecting nucleosomes (SWI/SNF/BRG1, BRM) or depositing histone variants (INO80/SRCAP). The ISWI and CHD family primarily mediate nucleosome maturation and spacing to promote chromatin formation post-replication, highly structured chromatin (ISWI), or transcriptional repression (CHD) (Clapier et al., 2017).

Mutations in these enzyme families results in aberrant gene expression that impinges on many cellular activities including DNA replication, DNA repair, as well as cell proliferation and differentiation. As indicated above, mutations in many of these family members lead to a wide range of NDD and symptoms (Table 1) with some of the more well-studied disorders being Coffin-Siris syndrome (CSS), Nicolaides-Baraitser syndrome (NCBS), CHARGE syndrome, and ATR-X syndrome. Moreover, it is becoming clear that mutations in multiple components of these remodeling complexes cause ID (Table 1) and can contribute to a spectrum of clinical phenotypes that is best illustrated by mutations in the SWI/SNF interacting partners (Bögershausen and Wollnik, 2018; van der Sluijs et al., 2019). The reader is referred to a number of recent reviews for detailed information of these different remodeler classes (Hota and Bruneau, 2016; Sokpor et al., 2017; Goodwin and Picketts, 2018; Alfert et al., 2019; Hoffmann and Spengler, 2019).

**TABLE 1:** ATP-dependent chromatin remodelers are a frequent cause of NDDs. List of NDD implicated genes which are incorporated into ATP-dependent chromatin remodeling complexes.

Family	Gene	Associated disease	References
SWI/SNF	ARID1A	ID, CSS	Tsurusaki et al. (2012); Kosho et al. (2013)
	ARID1B	ID, CSS, ASD	Hoyer et al. (2012); Santen et al. (2012); De Rubeis et al. (2014)
	ARID2	ID, CSS-like, NCBS-like	Shang et al. (2015); Bramswig et al. (2017)
	DPF2	ID, CSS-like	Vasileiou et al. (2018)
	PBRM	ASD	O’Roak et al. (2012b)
	SMARCA2	NCBS	Van Houdt et al. (2012); Tsurusaki et al. (2014b)
	SMARCB1	ID, CSS, Kleefstra	Kleefstra et al. (2012); Santen et al. (2013); Wieczorek et al. (2013)
	SMARCC1	ASD	Hormozdiari et al. (2015)
	SMARCC2	ID, ASD	Machol et al. (2019)
	SMARCE1	CSS-like	Tsurusaki et al. (2012); Kosho et al. (2013); Wieczorek et al. (2013)
	SMARCA4	CSS	Santen et al. (2013); Tsurusaki et al. (2014b)
	SOX11	ID, CSS-like	Tsurusaki et al. (2014a)
ISWI	BAZ1A	ID	Zaghlool et al. (2016)
	BAZ1B	Williams-Beuren syndrome	Lu et al. (1998); Peoples et al. (1998)
	BAZ2B	ID, ASD	Scott et al. (2020)
	BPTF	ID	Stankiewicz et al. (2017)
	SMARCA1	CSS-like, Rett syndrome-like, schizophrenia	Karaca et al. (2015); Homann et al. (2016); Lopes et al. (2016)
CHD	CHD2	Lennox-Gastaut syndrome, Doose Syndrome, Epileptic encephalopathy	Carvill et al. (2013)
	CHD3	Macrocephaly, ID, impaired speech/language	Snijders Blok et al. (2018)
	CHD4	Sifrim–Hitz–Weiss syndrome	Weiss et al. (2020)
	CHD5	ASD-like	Pisansky et al. (2017)
	CHD7	CHARGE syndrome, ASD	Vissers et al. (2004); Sanlaville et al. (2006); O’Roak et al. (2012b)
	CHD8	ASD	O’Roak et al. (2012a, b); De Rubeis et al. (2014); Neale et al. (2016)
INO80	INO80	Microcephaly, ID	Alazami et al. (2015)
	SRCAP	Floating-harbor syndrome	Hood et al. (2012)
	YY1AP1	ID	Guo et al. (2017)
ATRX	ATRX	ATR-X syndrome	Gibbons et al. (1995)

Here, we will review recent studies on ATRX to highlight the multiple biochemical functions chromatin remodeling proteins participate in, and the diverse set of mechanisms that, collectively, contribute to the complexity underlying the pathogenesis of NDD.

## Molecular Genetics of the ATR-X Syndrome

The ATR-X syndrome is a rare human congenital disorder with a wide range of symptoms that primarily affects males. Over 200 cases have been identified worldwide with and an estimated prevalence of <1–9/1,000,000 (Gibbons, 2006). Affected individuals display cognitive impairment typically described as severe ID, and many are non-verbal, capable of speaking or signing only a few words (Saugier-Veber et al., 1995; Guerrini et al., 2000). Originally, the presence of alpha thalassemia was used as a diagnostic tool to identify affected individuals, but there is variability in the hematological symptoms (Gibbons et al., 1995). The majority of patients are affected with microcephaly and skeletal malformations (Holmes and Gang, 1984; Carpenter et al., 1999). Muscle development is also impaired in most, leading to delayed motor development and hypotonia, while approximately one third of patients experience seizures (Lossi et al., 1999).

**FIGURE 2:**
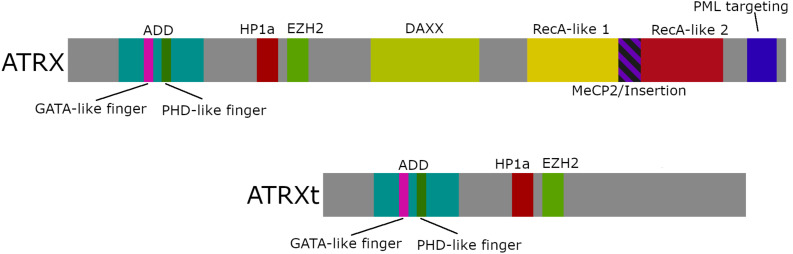
ATRX domain structure. Schematic diagram of full-length ATRX (282 kDa), truncated ATRX (ATRXt; 180 kDa), and locations of the key protein interaction domains. The two isoforms share an ADD domain, a HP1α binding motif, an EZH2 binding motif, while the ADD domain is comprised of a GATA-like zinc finger and a PHD-like zinc finger. The full-length polypeptide also contains a DAXX binding motif, a SNF2-ATPase domain comprising RecA-like lobes 1 and 2 separated by a linker region containing a MeCP2 binding motif (MeCP2/Insertion), and a PML targeting motif.

Although ATR-X syndrome patients present with a heterogeneous phenotype, the disease is caused by mutations in a single gene, the *ATRX* locus, which spans over 300 kbp on chromosome Xq13.3-21.1 (Gibbons et al., 1995, 2008; Picketts et al., 1996). The *ATRX* gene encodes two major trasncripts (Figure 2), one encoding the full length protein and a truncated isoform generated by an alternative splicing event that retains intron 11 and terminates translation prematurely (Garrick et al., 2004; Mitson et al., 2011). The full length transcript encodes a protein of 285 kDa in size while the shorter transcript generates a smaller truncated protein that is 180 kDa and lacks the ATP-dependent remodeling domain (Picketts et al., 1996; Garrick et al., 2004).

The *N*-terminus of the ATRX protein houses several motifs critical for its interaction with chromatin, including a heterochromatin protein 1 (HP1α) binding motif (PxVxL) (Lechner et al., 2005) and enhancer of zeste homolog 2 (EZH2) interaction domain (Cardoso et al., 1998), and the ATRX-DNMT3-DNMT3L (ADD) domain (Picketts et al., 1998; Xie et al., 1999). The ADD domain comprises a GATA-like zinc finger and a plant homeodomain (PHD)-like finger that targets the dual histone post translational modification (PTM), H3K9me3 and H3K4me0 (Argentaro et al., 2007; Dhayalan et al., 2011; Eustermann et al., 2011; Iwase et al., 2011). A region within the center of the polypeptide mediates death domain associated protein (DAXX) binding (Xue et al., 2003). Toward the *C*-terminus lies the highly conserved RecA-like lobes 1 and 2 that together are required for ATPase activity (Picketts et al., 1996), as well as mapped regions for interactions with the methyl-CpG-binding protein (MeCP2) (Nan et al., 2007) and the promyelocytic leukemia protein (PML) (Bérubé et al., 2008) (Figure 2).

The majority of ATR-X syndrome causing mutations are missense mutations mapping within the ADD (50%) and SNF2-like/helicase domains (30%) (Argentaro et al., 2007; Gibbons et al., 2008). To date, there has been a lack of genotype: phenotype correlations identified, although mutations within the ADD domain typically produce more severe psychomotor phenotypes compared to mutations in the SNF2-like/helicase domain (Badens et al., 2006).

It should also be noted that somatic mutations in the *ATRX* gene have been identified in a wide range of cancers that include pancreatic neuroendocrine tumors, gliomas, neuroblastomas, and sarcomas, which will not be discussed here but have been the focus of recent reviews (Watson et al., 2015; Dyer et al., 2017).

## Interacting Partners and Biochemical Functions

All functional studies indicate that ATRX is a heterochromatin interacting protein. It localizes to pericentromeric heterochromatin, telomeres, PML nuclear bodies, and physically interacts with the HP1 family (McDowell et al., 1999; Berube et al., 2000; Tang et al., 2004). Later work demonstrated that ATRX could be recruited to the heterochromatin histone mark, H3K9me3, either indirectly by its interaction with HP1 or recruitment by MeCP2, and directly by binding of the ADD domain to H3K9me3 that lies adjacent to unmethylated H3K4 (Berube et al., 2000; Bannister et al., 2001; Nan et al., 2007; Eustermann et al., 2011; Iwase et al., 2011). ATRX and DAXX were identified as interacting partners by two separate groups, one using ATRX co-IP experiments and the other a Flag-DAXX pull-down approach (Xue et al., 2003; Tang et al., 2004). Further characterization showed that most of the endogenous ATRX protein is in a 1 MDa complex with DAXX, while DAXX also fractionates in a 700 kDa complex independent of ATRX. Deletion mutants were used to demonstrate that the ATRX/DAXX interaction was mediated through the PAH domain of DAXX and a region between the ADD and SNF2 domains within ATRX (Tang et al., 2004). Both the ATRX/DAXX complex and recombinant ATRX protein had DNA or nucleosome stimulated ATPase activity which was impaired by patient mutations that localized to the ATPase domain (Xue et al., 2003; Tang et al., 2004). A mononucleosome disruption assay was used to demonstrate that the ATRX/DAXX complex could alter the DNAse I digestion pattern of the mononucleosome in the presence of ATP. The localization of the altered digestion pattern indicated that ATRX/DAXX disrupts DNA–histone interactions at the entry site of the nucleosome and does not alter nucleosome phasing. In addition, a triple-helix strand displacement assay was used to show that the ATRX/DAXX complex and ATRX alone had a DNA translocase property similar to the RSC and SWI/SNF complexes (Xue et al., 2003). More recent work has indicated that DAXX is an H3.3-specific histone chaperone that functions with ATRX to deposit the histone variant at pericentric and telomeric repeats, while DAXX functions independently of ATRX to repress retrotransposons (Lewis et al., 2010; Hoelper et al., 2017). In this regard, the ATRX/DAXX complex shows some similarities with the ISWI complex ACF and its interactions with the histone chaperone NAP1 (Gemmen et al., 2005; Torigoe et al., 2011). These properties could be used to reconstitute H3.3 containing nucleosomal arrays that might guide future *in vitro* biochemical studies to further define ATRX function during transcription or DNA replication (Peterson, 2009).

Indeed, in a series of papers ATRX, DAXX, and the histone variant H3.3 were shown to co-localize at telomeres where the ATRX/DAXX complex functions as a histone chaperone to deposit H3.3 into telomeric heterochromatin (Wong et al., 2009; Goldberg et al., 2010; Lewis et al., 2010). Further work showed that DAXX functions as the histone chaperone, that H3.3K9me3 deposition occurs in a replication-independent manner by the complex, and both H3.3 loading and heterochromatin organization by ATRX/DAXX is mediated by SUV39H1 and PML in PML-associated heterochromatin domains (Drané et al., 2010; Goldberg et al., 2010; Lewis et al., 2010; He et al., 2015; Udugama et al., 2015; Delbarre et al., 2017). Additionally, ATRX was shown to be critical for the formation of senescence-induced heterochromatin foci (SAHF) that help drive cancer cells into therapy-induced senescence (Kovatcheva et al., 2017). Finally, ATRX has been shown to bind to the *Xist* lncRNA to promote recruitment of the PRC2 repressive complex and facilitate stable heterochromatin formation of the silenced X-chromosome (Sarma et al., 2014). RNA binding remains an understudied role for ATRX, although several reports have shown a range of interactions with multiple lncRNAs including TERRA (telomeric repeat-containing RNA) (Chu et al., 2017; Nguyen et al., 2017), *ChRO1* in muscle (Park et al., 2018), and minor satellite RNAs at centromeric heterochromatin (Ren et al., 2020). These interactions are mediated through a unique *N*-terminal domain in ATRX to regulate differentiation, gene expression, DNA and histone methylation and chromatin compaction (Chu et al., 2017; Nguyen et al., 2017; Park et al., 2018; Ren et al., 2020).

A role for ATRX at heterochromatin was also strengthened by chromatin immunoprecipitation experiments that showed enriched ATRX binding at telomeres and centromeres. Interestingly, ATRX was also enriched at repetitive DNA elements while having a lower frequency of binding within gene bodies (Law et al., 2010). Further characterization showed that ATRX was prevalent at long terminal repeats of endogenous retrovirus sequences of family K (ERVK), at variable number tandem repeats (VNTRs) and at simple tandem repeats (Law et al., 2010). Many of the tandem repeats were GC-rich sequences that are predicted to form G-quadruplex secondary DNA structures (G4 DNA) including the telomeric repeats and some CpG islands. The formation of G4 DNA has been proposed to have important roles in the regulation of gene expression, as well as be prohibitive to DNA replication and transcription (Rhodes and Lipps, 2015; Valton and Prioleau, 2016; Varshney et al., 2020). *In vitro* studies confirmed that ATRX can bind to G4 DNA structures (Law et al., 2010). In addition, ATRX mutations have variable effects on α-globin expression including individuals with the same mutation. Law et al. (2010) demonstrate that one ATRX binding site lies within a GC-rich VNTR sequence 1 kb upstream of the HBM gene. The authors demonstrate a positive correlation in ATR-X patients such that increasing VNTR repeat size increases the severity of the α-thalassemia as measured by the level of HbH inclusions in red blood cells. Since the sequence is a GC-rich VNTR that is predicted to form G4 quadruplexes, it was inferred that increasing repeat size increases the probability to form G4 DNA that subsequently alters HBM expression.

The ATRX protein was also shown to co-purify with the MRE11-RAD50-NBS1 (MRN) complex, an active player in the processing of DNA double strand breaks (DSB) that suggested ATRX was critical to maintain genome integrity (Leung et al., 2013). Consistent with this finding, ATRX knockdown studies in HeLa cells resulted in defects in mitotic progression and micronuclei formation from altered chromosome condensation and centromeric cohesion (Ritchie et al., 2008). Other studies indicated that ATRX loss impaired replication fork progression during S-phase resulting in telomere fragility, increased DSB, and mitotic catastrophe (Huh et al., 2012, 2016; Leung et al., 2013; Watson et al., 2013).

The ATRX-DAXX-H3.3 complex is critical for this heterochromatic formation and subsequent maintenance (Law et al., 2010; Eid et al., 2015; He et al., 2015; Udugama et al., 2015). H3.3 within telomeric regions is targeted for trimethylation on its K9 residue (He et al., 2015; Udugama et al., 2015). H3.3K9me3 recruits more ATRX-DAXX-H3.3 complexes, which in turn will deposit H3.3, creating a positive feedback loop required for maintaining telomere structure (Udugama et al., 2015). Failure to establish proper structure will reduce telomere integrity and result in an increase of non-coding telomeric transcript expression (He et al., 2015; Udugama et al., 2015).

The eclectic properties of the ATRX protein do not make it intuitively obvious how an aberration of these functions can result in a neurodevelopment disorder with cognitive deficits. In the remaining section, we discuss the characterization of mouse models and the insights they have provided into the pathophysiology of ATR-X patients and, more generally, the complex etiology of NDDs caused by defective epigenetic regulators.

## Delineating Pathophysiological Mechanisms of the ATR-X Syndrome

### Functional Effects of Patient Mutations and Generation of Animal Models

One of the first questions addressed was do patient mutations affect protein stability and function? Immunoblots of extracts from patient-derived EBV-transformed B-lymphocytes showed significantly reduced levels of ATRX protein from all patients tested (McDowell et al., 1999; Cardoso et al., 2000). Interestingly, in patients with early premature stop codons (e.g., p.Arg37X), translation was initiated from an internal methionine that produced a smaller truncated protein at ∼30% levels leading to a milder phenotype (Howard et al., 2004; Abidi et al., 2005; Basehore et al., 2015). Utilizing recombinant proteins, other studies demonstrated that mutations within the ATPase domain attenuated ATPase activity but did not reduce it, while mutations in the ADD domain or the PML-targeting domain reduced localization to chromocenters and PML nuclear bodies, respectively (Cardoso et al., 2000; Bérubé et al., 2008). *Atrx*-null mutations in mice show defective extraembryonic trophoblast development and die embryonically at ∼E9.5 (Garrick et al., 2006). Collectively, these studies indicate that ATR-X syndrome causing mutations are functional hypomorphs, while more severe mutations are not found and are presumably non-viable.

Several different ATRX-deficient mouse lines have been generated and used for functional characterization. The most widely used model is a floxed allele in which loxP sites flanked exon 18 which encodes the ATP-binding pocket (Bérubé et al., 2005; Garrick et al., 2006). These animals have been crossed with several different tissue-specific Cre driver lines to inactivate ATRX in skeletal muscle progenitors (Huh et al., 2012), Sertoli cells (Bagheri-fam et al., 2011), osteobalsts (Solomon et al., 2013), chondrocytres (Solomon et al., 2009), the retina (Medina et al., 2009; Lagali et al., 2016), and the developing forebrain (Bérubé et al., 2005) among others. A second transgenic line (*Atrx^Δ*E*2^*) was developed by deleting exon 2 and replacing it with a SA-IRES-β-geo cassette (Nogami et al., 2011; Shioda et al., 2011). This mutation was meant to mimic the p.Arg37X mutation and make an *N*-terminally truncated ATRX protein by initiating translation from an internal methionine codon (Howard et al., 2004; Abidi et al., 2005). Both of these models will be discussed in more detail in the following sections. Finally, an overexpression transgenic line was created with the *ATRX* cDNA under control of a CMV enhancer/β-actin promoter which resulted in growth retardation, neural tube defects and a high incidence of embryonic lethality demonstrating the importance of ATRX dosage to normal development (Berube et al., 2002).

While each of these models has provided valuable insight into disease mechanisms (as highlighted below), the field still awaits a model whereby a single nucleotide variant is introduced into the *ATRX* gene to recreate a known patient mutation, such as the common p.Arg246Cys mutation within the ADD domain.

### Replication Stress Impairs Progenitor Expansion Resulting in Microcephaly

Microcephaly is common to many NDDs and has also been observed in mouse models that deleted other genes encoding chromatin remodeling proteins (Ronan et al., 2013). Most ATR-X patients develop postnatal microcephaly and, in instances where CT or MRI scans have been performed, mild cerebral atrophy was detected. Similarly, three patient autopsy reports also described that the brains were smaller in size (Gibbons, 2006).

The first indication that ATRX may be critical for cell growth came from co-culture experiments of embryonic stem cells (ESC) from control or *Atrx*-null cells. This experiment demonstrated that the *Atrx*-null cells were underrepresented after 4-days of co-culture. Flow cytometry was used to examine cell cycle distribution but no differences were observed suggesting that cells may have transitioned to a slower cycling, differentiated cell type (Garrick et al., 2006). Given that ATRX has high expression in the developing forebrain, the *Atrx*^*fl/fl*^ line was next crossed with the forebrain-specific Foxg1-Cre line (*Atrx^*Foxg*1*Cre*^*) that initiates Cre expression in the developing telencephalon at ∼E8.5 (Hébert and McConnell, 2000). Loss of ATRX caused a 25–30% reduction in cell number with a noticeably smaller neocortex and hippocampus including almost a complete absence of the dentate gyrus that likely contributed to early postnatal lethality (Bérubé et al., 2005). Similar to ESC co-culture experiments, BrdU-pulse labeling experiments suggested no differences in the proportion of cycling cells. However, there was a dramatic increase in the number of TUNEL+ cells leading to a reduction in the number of neurons that reached the cortical layers (Bérubé et al., 2005). Similarily, the *Atrx^Δ*E*2^* mice were smaller and also showed brain hypocellularity, although to a milder extent (Nogami et al., 2011). *Atrx* inactivation in Sertoli and muscle cells, also showed a significant impact on the growth of the tissue (Bagheri-fam et al., 2011; Huh et al., 2012). However, a retina progenitor cell cKO only had a limited effect on the size of the mature tissue suggesting that defects in cell cycle progression lead to significant hypocellularity among tissues that require a rapid expansion over a narrow developmental timeframe (Medina et al., 2009).

Although not initially observed, delayed cell cycle progression through both S- and G2/M phases was later observed in other studies (Ritchie et al., 2008; Watson et al., 2013; Huh et al., 2016). For G2/M, the progression from prometaphase to metaphase was prolonged and associated with sister chromatid cohesion and congression defects that impaired proper separation at anaphase leading to DNA bridges and micronuclei (Ritchie et al., 2008). Evidence for DNA bridges and micronuclei in *Atrx^*Foxg*1*Cre*^* mice were also detected by high magnification microscopy at the apical surface on cortical sections of the neuroepithelium where cortical progenitors complete mitosis. Interestingly, a recent study has also demonstrated that ATRX promotes telomere cohesion between sister telomeres to mediate the repair of DNA DSB (Lovejoy et al., 2020).

Defects in S-phase were observed using BrdU-pulse chase flow cytometry experiments where a delay from G1 to S-phase and also from G2/M to the following G1 phase was identified (Huh et al., 2012). Co-labeling experiments demonstrated that ATRX associated with replicating chromatin during mid-late S-phase and cytological analysis showed a high prevalence of genomic instability that was enriched at telomeres and pericentromeric heterochromatin (Huh et al., 2012; Watson et al., 2013). Moreover, treatment with a compound that binds and stabilizes G4 DNA increased the number of telomere dysfunction induced foci (TIFs) and decreased cell viability suggesting that G4 DNA formation was the main cause of replicative stress (Watson et al., 2013). Other studies indicate that replication stress at telomeres may be mediated by increased TERRA transcription (Nguyen et al., 2017). TERRA levels are tightly regulated and critical for both telomere formation, replication and maintenance (Bettin et al., 2019). However, when TERRA levels increase, as shown for ATRX-null cells, it enhances R-loop (RNA-DNA hybrid) formation and G4 DNA stabilization, each of which increase replication fork stalling and collapse that then induces homology directed repair (HDR) and TIFs (Nguyen et al., 2017). The regulation of R-loops has also been proposed for other proteins that interact with G4 DNA during replication and/or at telomeres (Zhou et al., 2014; Ribeiro de Almeida et al., 2018; Toubiana and Selig, 2018; Maffia et al., 2020). It should also be mentioned here that somatic *ATRX* mutations, and to a lesser extent H3.3 and DAXX mutations, are prevalent in cancers characterized by ALT (alternative lengthening of telomeres), a HDR mechanism to maintain telomere length that is normally suppressed by ATRX (Heaphy et al., 2011; Lovejoy et al., 2012; Schwartzentruber et al., 2012; Pickett and Reddel, 2015; Verma and Greenberg, 2016).

Another indicator of replicative stress as a major impediment to growth of *Atrx*-null cells was demonstrated by studies showing an increased sensitivity to hydroxyurea, enhanced DSBs, and the use of DNA fiber analysis to show increased stalled replication forks and reduced origin firing (Leung et al., 2013; Clynes et al., 2014; Huh et al., 2016). Mechanistically, ATRX physically interacts with the MRN complex where it is thought to block HDR at stalled replication forks to allow for fork restart after the G4 DNA is resolved (Clynes et al., 2014). Indeed, one group demonstrated that fork protection could be restored by treatment with an Mre11 exonuclease inhibitor (Huh et al., 2016). This study also suggested that hyperactivation of poly (ADP-ribose) polymerase-1 (Parp-1) during neurogenesis may function as a compensatory mechanism to protect stalled replication forks from collapse and HDR, thus dampening the extent of cell loss during neurogenesis.

During mouse cortical development, the cortical layers are formed in sequential fashion from a pool of neural progenitor cells (NPC) that must continue to proliferate to maintain the pool size. Alterations in NPC proliferation depletes the pool often resulting in altered cell lamination typically observed as a reduction in upper layer neurons. For *Atrx^*Foxg*1*Cre*^* mice, the most proliferative NPCs that ultimately would become upper layer neurons are more susceptible to incur replication-induced DNA damage. Frequently the resulting genomic instability will occur at telomeres and pericentromeric heterochromatin, but it could also occur at other genomic regions that can form G4 DNA or similar secondary DNA structures that induce replication fork stalling and collapse. Accumulation of sufficient damage further leads to their demise and decreases neuron production and brain size. Indeed, the *Atrx^*Foxg*1*Cre*^* forebrain is reduced in size with a compromised production of upper layer neurons (Ritchie et al., 2014; Huh et al., 2016). Similar results have been observed in mice lacking CHD4 and SMARCA5 where the NPCs either fail to progress through the cell cycle or incur significant DNA damage, respectively, prior to undergoing apoptosis (Alvarez-Saavedra et al., 2014, 2019; Nitarska et al., 2016).

The SWI/SNF complex is also critical for brain development but utilizes different mechanisms than ATRX. The SWI/SNF complex is required during early neurogenesis for differentiation from radial glial progenitor cells into intermediate progenitor cells. This switch from a neural stem cell to an NPC is accompanied by the fundamental shift from the npBAF to nBAF complex, which involves the substitution of three subunits (BAF45, BAF53, and BAF55). Failure to switch leads to increased cell death, a small progenitor pool, and failure to further differentiate (Lessard et al., 2007; Wu et al., 2007; Bachmann et al., 2016). Interestingly, while SMARCA5 loss hampers NPC proliferation, loss of its ISWI ortholog SMARCA1 fails to repress expression of proliferation genes resulting in delayed neuronal differentiation and a larger brain (Yip et al., 2016). Taken together, these examples highlight the importance of chromatin remodeling proteins to NPC homeostasis and provide insight into the multitude of mechanisms at work often resulting in a similar phenotype.

### Transcriptional Deficits Associated With ATRX Mutations

Chromatin remodeling proteins were first identified as transcriptional coactivators and they continue to be implicated in the regulation of many genes. Since its identification, ATRX has also been presumed to regulate gene transcription. While there is a good level of understanding regarding how ATRX maintains genomic stability through the regulation of tandem repeats, telomeres and pericentromeric heterochromatin, the identification of direct transcriptional targets has proven more challenging. Initial ChIPseq experiments suggested that ATRX was bound at few promoters, gene bodies and regulatory elements (Law et al., 2010). Further work has suggested that ATRX binding may differ between tissues to ensure proper silencing of repetitive elements located near or within expressed genes in that particular tissue (Nguyen et al., 2017). Consistent with this idea, ATRX ChIPseq analysis of NPCs demonstrated a higher enrichment of binding sites at gene regulatory elements compared to what was observed in mouse ESCs suggesting that more genes may be under direct ATRX regulation within the brain (Law et al., 2010; He et al., 2015; Danussi et al., 2018). Another contributing factor to differential target gene expression is represented by ATRX effects on α-globin gene expression. Mutational analysis identified >15 ATR-X patients with the identical missense change (p.Arg246Cys), yet they showed a variable degree of hemoglobin H inclusions in blood samples, indicative of differing levels of α-globin expression (Gibbons et al., 1997). Repression of α-globin expression was dependent on the size of a GC-rich VNTR located within the globin gene cluster (Law et al., 2010). A second factor driving the tissue specificity and the variable effects was the formation of R-loops caused by the transcription of the GC-rich VNTR sequences (Nguyen et al., 2017). The larger sequences generate increased R-loops and G4 DNA structures that normally recruit ATRX to re-establish the normal chromatin structure. In the absence of ATRX the R-loop/G4 DNA is not resolved effectively which then impedes both replication and transcription processes (Nguyen et al., 2017). The slight stochastic nature of these effects likely also dampens readouts of differential expression from RNAseq experiments thereby raising the need for a scRNAseq approach in future studies.

Gene expression analysis of control and *Atrx^*Foxg*1*Cre*^* cortical samples at two timepoints (E13.5, P0.5) identified 202 and 304 differentially expressed genes (DEGs; ±1.5-fold change), respectively, with almost two-thirds of genes upregulated (Levy et al., 2008). Among these, 27 were common to both datasets including the downregulation of several ancestral pseudoautosomal region (aPAR) genes (Csfr2a, Dhrsxy, Cd99, and Asmtl) (Levy et al., 2008, 2014). In mouse, the aPAR genes are located in subtelomeric regions and contain potential G4 DNA sequences. Each gene analyzed had enriched histone H3.3 and ATRX binding within their gene body and showed reduced H3.3 levels when ATRX was absent (Levy et al., 2014). Interestingly, these intragene G4 DNA sequences also showed increased binding of RNA pol II in *Atrx^*Foxg*1*Cre*^* samples suggesting that transcription becomes impeded at these regions within the gene leading to reduced expression. The authors extended this finding to *Nlgn4*, a gene encoding a post-synaptic cell adhesion molecule implicated in ASDs (Jamain et al., 2003; Laumonnier et al., 2004). This result conflicted with the study on R-loop formation which found no differences in RNA pol II loading or histone modifications across genes containing the GC-rich repeats (Nguyen et al., 2017).

Other downregulated genes from this analysis include *Gbx2*, *NeuroD4*, *Wif1*, *Nxph1*, *Nxph2*, and *Mbp*, each of which could contribute to cognitive deficits observed in patients but require further analysis to asses their contribution to the phenotype (Levy et al., 2008, 2014). In a similar experiment in the retina, 173 DEGs were identified with two-thirds upregulated (109 genes) and one-third (64 genes) downregulated (Lagali et al., 2016). Most of these genes were involved in the regulation of glutamate activity, ion channel regulation or encoded neuroprotective peptides, with four shown to be also dysregulated in the cortex (*Csf2ra*, *Cbln4*, *Syt13*, and *Nlgn4*). Each of these studies showed that the mutant samples had only small numbers of genes with large changes in gene expression and, while some downregulated genes may impede transcriptional elongation, this mechanism may not be universal, particularly as it pertains to upregulated genes.

However, other indirect mechanisms have been explored to explain transcriptional dysregulation, particularly a loss of repression. The ATRX/DAXX complex is critical for loading H3.3 at telomeres and pericentromeric heterochromatin (Goldberg et al., 2010; Wong et al., 2010). Research over the last few years has expanded this regulation to include H3.3 deposition at endogenous retroviral elements, regions associated with imprinted genes, and some CpG islands (Elsässer et al., 2015; He et al., 2015; Sadic et al., 2015; Voon et al., 2015). At the telomere, the loss of ATRX affected the transcription of telomeric DNA and the non-coding RNA TERRA, although studies conflict on whether levels increase or decrease (Eid et al., 2015; Nguyen et al., 2017). Surprisingly, TERRA was also shown to bind to an additional ∼4,000 binding sites aside from the telomere where it co-localized with ATRX (Chu et al., 2017). Many of these sites were within introns and comprised GA repeats, however, depletion of TERRA usually resulted in downregulation while ATRX depletion increased expression (Chu et al., 2017). While it remains to be determined how this might affect neuronal gene expression, ATRX has been shown to interact with other lncRNAs including *Xist* to facilitate PRC2 silencing and *ChR01* that is required for heterochromatin reorganization in differentiating muscle cells (Sarma et al., 2014; Park et al., 2018). Indeed, ATRX binding to lncRNA or R-loops may be a key mechanism mediating transcriptional repression of specific target genes.

Histone H3.3 ChIPseq studies have also shown that it is enriched at the intracisternal A-particle endogenous retroviral elements (IAP/ERVs), which account for almost half of all mutation causing ERV insertions (Maksakova et al., 2006; Elsässer et al., 2015). Moreover, H3.3 deposition at these sites requires ATRX/DAXX to facilitate H3K9me3 and repression while depletion of ATRX, DAXX, or H3.3 results in reduction of the H3K9me3 mark and IAP/ERV derepression (Elsässer et al., 2015; He et al., 2015; Voon et al., 2015). In mouse ESCs, ERV derepression affected the expression of neighboring genes in a minority of cases with most genes neutral to ERV derepression. It raises the question of whether or not this type of derepression would affect many genes or occur rapidly within a post-mitotic neuron, and thus, function as a major effector in dysregulated gene expression in *Atrx*-null neurons. In this regard, a related study using cultured post-mitotic neurons demonstrated that the ADD domain can also bind the H3K9me3S10ph dual histone mark (Noh et al., 2015). This histone mark is rapidly induced by neuronal depolarization where it appears at centromeric and pericentromeric heterochromatin co-localized with ATRX to repress transcription of non-coding centromeric minor satellite sequences (Noh et al., 2015). While it is unclear what the impact of increased centromeric minor satellite transcription would have on disease pathology, it remains to be determined whether this dual mark affects activity-dependent transcription of genes mediating learning or memory.

It was also demonstrated that ATRX was bound to 56 CpG islands which was unexpected since they are often associated with active chromatin, typically promoters (Voon et al., 2015). However, these CpG islands were associated with H3K9me3, almost half were methylated and many corresponded to imprinted loci often residing in intragenic regions within a transcriptional unit (Voon et al., 2015). Indeed, in all cases examined ATRX was bound to the silenced imprinted allele which became reactivated in ATRX KO cells (Voon et al., 2015). This study contrasted somewhat with an independent report in which ATRX was recruited by MeCP2 to silence the active allele of several imprinted genes in the developing telencephalon (Levy et al., 2014). The difference in these studies may reflect differential regulation of imprinting loci in ESCs versus differentiating NPCs. Perhaps the most compelling example of derepression came from a study with the *Atrx^Δ*E*2^* mice (Shioda et al., 2018). In this model, the authors identify a small list of 31 DEGs in the adult hippocampus but with most genes (23/31) downregulated (Shioda et al., 2018). Among the upregulated genes was an imprinted gene from the lymphocyte-regulated gene family, *Xlr3b*. Although *Xlr3b* had widespread expression across many tissues, it was only upregulated in the brain. Further work showed that ATRX bound to a G4 DNA sequence within the CpG island of the *Xlr3b* gene where it normally interacted with DAXX and H3.3 and recruited DNMT1 and DNMT3 to silence the gene. The subsequent overexpression of *Xlr3b* in the *Atrx^Δ*E*2^* mice was shown to produce a protein that localized to dendritic RNA granules where it interacted with ribonucleoproteins, dynein proteins and the RNA-binding protein, TIA1, to regulate mRNA transport (Shioda et al., 2018). One of the targets identified was the mRNA for CAMK II-α which they had previously shown to be deregulated in these animals. Excitingly, they also showed that the G-quadruplex-binding ligand 5-aminolevulinic acid (5-ALA) was able to decrease RNApol II occupancy and *Xlr3b* expression in the *Atrx^Δ*E*2^* mice, although methylation of the G4 DNA sequence within the CpG island was not affected. It seems that formation or stabilization of this G4 DNA sequence is required to activate the *Xlr3b* gene and that ATRX normally prevents this by facilitating heterochromatin formation. In this regard, mapping of G4 DNA sequences have shown an enriched number in gene regulatory elements where many function to increase transcription when stabilized (Hänsel-Hertsch et al., 2016). While G4 DNA stabilization occurs in the *Atrx^Δ*E*2^* mice, further work is required to explain how 5-ALA represses Xlr3b transcription when it should stabilize the G4 DNA. Regardless, the derepression of G4 DNA within CpG islands and/or other regulatory elements is an exciting mechanism that can explain DEG upregulation, particularly when coupled with the finding that ATRX binding is increased at regulatory elements in NPCs compared to ESCs. Collectively, the derepression of tandem repeats, retrotransposable elements and G4 quadruplexes can all function to impinge on neuronal function.

### Morphological, Behavioral, and Cell Non-autonomous Deficits

We have discussed global effects on DNA replication and transcription that occur in the absence of ATRX in the previous two sections. In this section, we review the morphological and functional repercussions of these deficits. Aside from being reduced in size, the *Atrx^*Foxg*1*Cre*^* mice had a normal cortical morphology with proper lamination although a reduction of upper layer neurons (Bérubé et al., 2005; Ritchie et al., 2014; Huh et al., 2016). The reduction in upper layer neurons may also contribute to the partial agenesis of the corpus callosum observed in some patients (Gibbons, 2006). The hippocampus was also reduced in size while the dentate gyrus consisted of only a few disorganized cells. Behavior analysis was not performed due to the early postnatal lethality, although female heterozygous mice showed impairment in spatial, contextual fear, and novel object recognition memory (Tamming et al., 2017).

The *Atrx^Δ*E*2^* mice also had smaller brains but no differences in cell density within layers II/III of the prefrontal cortex (PFC) or hippocampus (Shioda et al., 2011). Examination of dendritic spines in the PFC showed that the *Atrx^Δ*E*2^* mice had similar numbers but fewer mature spines and many more, thin, long immature spines (Shioda et al., 2011). Behavioral analysis indicated that the mice have impaired contextual fear memory (fear conditioning test), spatial memory (Y-maze), but not anxiety behaviors (Nogami et al., 2011; Shioda et al., 2011). Electrophysiology studies in hippocampal slices demonstrated reduced NMDAR-dependent long term potentiation (LTP) evoked by high stimulation frequency in hippocampal CA1 neurons which was mediated by increased CAMK2A and GluR1 phosphorylation (Nogami et al., 2011). This was in contrast to a later article by the same group that showed phosphorylated CAMK2A levels were reduced in the *Atrx^Δ*E*2^* mice while 5-ALA restored the levels at the synapse and the phosphorylation levels (Shioda et al., 2018). A recent article examining hippocampal function using CAMKII-Cre mice to inactivate *Atrx* in postnatal excitatory forebrain neurons demonstrated reduced paired-pulse facilitation and LTP in proximal and distal apical dendrites of CA1 synapses (Gugustea et al., 2019). This represented the first study of mice in which *Atrx* has been inactivated after neurogenesis and it will be interesting to ascertain the full characterization of these mice.

Studies of the retina have also provided useful information into ATRX function. Many ATR-X patients have visual problems although this has been an under-appreciated aspect of the phenotype (Medina et al., 2009). Inactivation of ATRX in retinal progenitors *Atrx^*Pax*6*Cre*^* resulted in a slight reduction in retina size and a specific reduction of interneurons, namely amacrine and horizontal cells (Medina et al., 2009). Surprisingly, *Atrx^*Pitf*1*aCre*^* mice that ablates ATRX in a bi-potential progenitor that generates amacrine or horizontal cells did not recapitulate the phenotype, while inactivation with a bipolar cell specific Cre driver (*Atrx^*Vsx*2*Cre*^*) did not affect bipolar cell survival but did result in reduced amacrine and horizontal cells suggesting that interneuron survival was a cell non-autonomous effect (Lagali et al., 2016). Additional characterization of these mice showed that the bipolar axons were mislocalized within the inner plexiform layer and many had axonal swellings or tortuous paths to their targets. Gene expression analysis identified alterations in the glutamate pathway, ion channel regulation and altered expression of neuroprotective peptides. Altered axonal pathfinding was also observed in Drosophila XNP mutants, the homolog to the ATPase domain of ATRX (Sun et al., 2006). It will be important to further explore in greater detail whether axonal pathfinding is also altered within forebrain or hippocampal neurons.

## Perspectives

**FIGURE 3:**
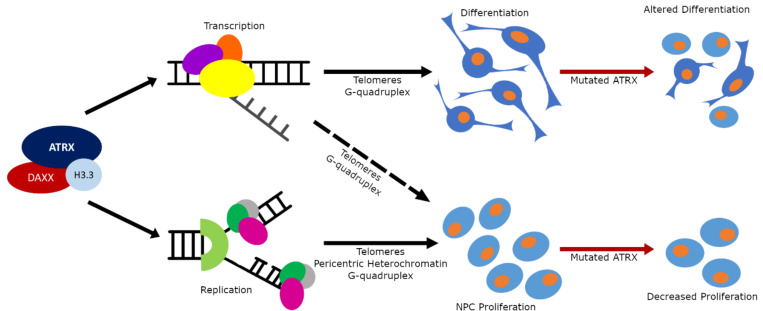
The multiple functions of the ATRX protein. Schematic diagram of ATRX functional influence on brain development and its contribution to NDDs. Normally ATRX utilizes its chromatin remodeling activity to (1) influence transcription and DNA replication in heterochromatic regions to control the rate of proliferation in the neuronal progenitor cell population (bottom arm); and (2) to influence transcription in heterochromatic regions to control differentiation processes (top arm). When ATRX is mutated the cellular proliferation rates in progenitors is slowed resulting in a smaller progenitor population; and the differentiation processes are altered resulting in either dysfunctional cellular morphology or complete absence of specific cell types.

Studies to date have indicated that ATRX has multiple roles during forebrain development that can contribute to the phenotype of ATR-X patients. It functions mainly as a heterochromatin interacting protein acting to ensure that repetitive DNA is properly packaged and organized into heterochromatin. We have highlighted how aberrations in heterochromatin maintenance leads to genomic instability and replication stress that impairs NPC expansion leading to a microcephalic brain (Figure 3). The loss of ATRX also affects gene expression typically resulting in increased gene derepression but also downregulation. It remains to be teased apart which targets are direct versus indirect, and when disrupted expression hampers neuronal function. It is likely that inactivation of ATRX in postmitotic neurons, following neurogenesis and lamination, will help define a role for ATRX target genes in altered synaptic activity and/or synaptic plasticity underlying cognitive impairment. Moreover, the contribution of other central nervous system cell types to the phenotype have not been explored. ATRX is expressed in glia and oligodendrocytes which are known to intimately communicate with neurons to mediate function, as shown recently in *Drosophila* glial ATRX dependent ensheathment of sensory neurons, for normal dendritic arborization and stimulus processing (Yadav et al., 2019). Intriguingly, MRI studies on ATR-X patients showed severe glial defects and white matter disruption, further stressing the need for research in this area (Wada et al., 2013; Lee et al., 2015). Importantly, a further understanding of ATRX function and its aberrant molecular pathways are required before potential treatments can be explored. In this regard, treatment with 5-ALA has shown promise in one animal model and it is being investigated in Japanese patients (T. Wada, personal communication). ATRX is but one of many different chromatin remodeling proteins mutated in NDDs but it serves to demonstrate how complex these disorders are and how widely chromatin remodelers impact cellular activities.

## Author Contributions

ST and DP wrote and edited the manuscript together. ST generated the figures and table. Both authors contributed to the article and approved the submitted version.

## Conflict of Interest

The authors declare that the research was conducted in the absence of any commercial or financial relationships that could be construed as a potential conflict of interest.

## Footnotes

1 https://sysid.cmbi.umcn.nl/
